# Intraoperative OCT for Lamellar Corneal Surgery: A User Guide

**DOI:** 10.3390/jcm12093048

**Published:** 2023-04-22

**Authors:** Antonio Moramarco, Natalie di Geronimo, Matteo Airaldi, Lorenzo Gardini, Francesco Semeraro, Danilo Iannetta, Vito Romano, Luigi Fontana

**Affiliations:** 1 Ophthalmology Unit, Department of Medical and Surgical Sciences (DIMEC), Alma Mater Studiorum—University of Bologna, 40126 Bologna, Italy; 2 Ophthalmology Unit, IRCCS Azienda Ospedaliero-Universitaria di Bologna, 40126 Bologna, Italy; 3 Eye Unit, ASST Spedali Civili di Brescia, Piazzale Spedali Civili, 1, 25123 Brescia, Italy; 4 Eye Unit, Department of Medical and Surgical Specialties, Radiological Sciences and Public Health, University of Brescia, Viale Europa 15, 25123 Brescia, Italy

**Keywords:** anterior segment imaging, artificial intelligence, multimodal imaging, ophthalmic imaging, optical coherence tomography, DALK, corneal transplantation, endothelial keratoplasty, mushroom PK

## Abstract

Intraoperative OCT is an innovative and promising technology which allows anterior and posterior segment ocular surgeons to obtain a near-histologic cross-sectional and tomographic image of the tissues. Intraoperative OCT has several applications in ocular surgery which are particularly interesting in the context of corneal transplantation. Indeed, iOCT images provide a direct and meticulous visualization of the anatomy, which could guide surgical decisions. In particular, during both big-bubble and manual DALK, the visualization of the relationship between the corneal layers and instruments allows the surgeon to obtain a more desirable depth of the trephination, thus achieving more type 1 bubbles, better regularity of the plane, and a reduced risk of DM perforation. During EK procedures, iOCT supplies information about proper descemetorhexis, graft orientation, and interface quality in order to optimize the postoperative adhesion and reduce the need for re-bubbling. Finally, mushroom PK, a challenging technique for many surgeons, can be aided through the use of iOCT since it guides the correct apposition of the lamellae and their centration. The technology of iOCT is still evolving: a larger field of view could allow for the visualization of all surgical fields, and automated tracking and iOCT autofocusing guarantee the continued centration of the image.

## 1. Intraoperative OCT: Technology and Characteristics

Anterior segment OCT (AS-OCT) was described for the first time in 1994 by Izatt et al. as an essential tool for the clinical diagnosis and follow up of many corneal pathologies [1]. Thanks to its high level of resolution, AS-OCT provides a near-histologic cross-sectional and tomographic image of the tissues, allowing for the detailed evaluation of clinical conditions, and is able to impact medical and surgical decisions. The introduction of this technology into the operating room was a natural consequence considering its several potential applications during both anterior and posterior segment surgery. Indeed, intraoperative OCT (iOCT) provides immediate feedback on the tissues’ anatomic configuration and could potentially directly guide surgical manipulations.

Standard OCT systems were unsuitable for a surgical setting due to their large dimensions and the traditional sitting position required for image acquisition, which was not practical for supine patients. The introduction of portable OCT systems allowed for the introduction of this tool into the operating room. The first two iOCT systems developed were the Bioptigen EnVisu (Bioptigen, Research Triangle Park, NC/Leica, Wetzlar, Germany) and the Optovue iVue (Optovue, Fremont, CA, USA) [2,3,4,5,6,7]. These systems were available in different configurations such as handheld, externally mounted, and microscope-mounted. Handheld imagine acquisition, although characterized by excellent image quality, is limited by potential motion artifacts, which could delay image capture and affect the quality of the resulting frames. In order to surpass these limitations, microscope-mounted systems were developed. These systems provided better stability; moreover, foot-pedal control of the microscope allowed for control of the probe location, with enhanced image reproducibility [8,9].

A major advance was achieved with the introduction of microscope-integrated OCT (MIOCT), which could finally enable the acquisition of real-time intraoperative OCT sections and the visualization of the instrument–tissue interaction [10,11]. Two of the currently most diffuse systems are the Zeiss OPMI LUMERA 700 (Carl Zeiss Meditec, Inc., Oberkochen, Germany) and the Leica Proveo 8 (Leica Microsystems, Wetzlar, Germany). Similar to the microscope-mounted systems, an MIOCT can be controlled by a foot pedal; furthermore, a heads-up display provides a combined visualization of the surgical field and the OCT data stream.

At first, iOCT was employed in vitreoretinal surgery, focusing on macular hole [8,12,13], vitreomacular traction [14,15], epiretinal membrane [16,17], and retinal detachment surgery [18,19]. More recently, it has been applied to glaucoma surgery [20], implantable collamer lens (ICL) implantation [21], cataract surgery [22], and corneal transplantation [23,24]. Concerning corneal surgery, its application is mainly related to lamellar surgery, both anterior and endothelial. Indeed, iOCT allows for a better visualization of the corneal layers, an evaluation of the instrument’s depth during anterior lamellar surgery, and confirmation of the complete adhesion of the lamella during endothelial surgery.

In this narrative review, we describe the application of iOCT during different types of corneal lamellar surgery and provide a useful and complete guide for both novice and expert corneal surgeons.

## 2. Intraoperative OCT Applications for Lamellar Corneal Surgery

### Guiding Big-Bubble Deep Anterior Lamellar Keratoplasty (BB-DALK)

Deep anterior lamellar keratoplasty (DALK) is the gold standard for the treatment of diseases of the anterior cornea, such as corneal ectasia, anterior stromal leucomas, and stromal dystrophies. In terms of improved visual acuity, DALK is associated with better outcomes than penetrating keratoplasty thanks to lower incidences of post-operative astigmatism and rejection [25,26]. Moreover, this technique does not require an open-sky approach, thus limiting possible risks and complications associated with the surgery [27,28].

There are several applications of iOCT, aimed mostly at avoiding the conversion to PK. First, it allows for the assessment of the corneal and anterior segment architecture, thus determining corneal thickness and regularity, and for the detection of the presence of anomalies such as Descemet’s membrane (DM) rupture or peripheral anterior synechiae. The definition of corneal anatomy aids in making decisions about the depth of trephination, which can be identified by a vertical hyperreflective band along the anterior stroma in the peripheral cornea, where the cut was made. In their study, Santorum et al. suggested an intended stromal bed of 150 μm within the posterior corneal surface, measured with the built-in caliper tool of the intraoperative OCT software (InVivoVue, IVV 2.18, Lumivero. Denver, CO, USA) [29]. However, in all their eyes, they needed to further extend the groove after the first trephination in order to obtain the desirable depth. Usually, we establish the depth of trephination based on the full thickness measured on the iOCT. The aim is to leave a residual stromal thickness of about 100 μm and thus reach the pre-Descemetic plane. The PIONEER study detected an incidence of further dissection after the initial trephination in 55.6% of cases, determined by an evaluation made by the surgeon in light of an iOCT scan [9].

**Figure 1:**
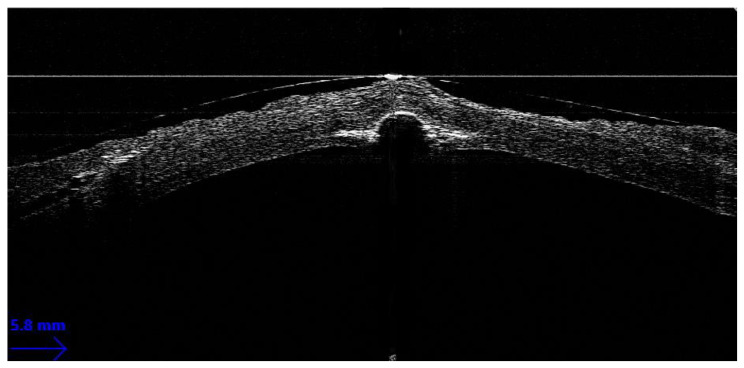
Seagull wing sign.

In BB-DALK surgery, the second step is the insertion of a cannula or needle into the stroma to create an air bubble. iOCT allows for the real-time visualization of the instrument, which can aid in guiding the insertion to the desirable depth and in the right direction. However, the visualization of the layers under the cannula can be masked by the hyperreflectivity of the instrument itself, hampering the correct assessment of the thickness of the tissue. In order to obtain the measurement of the residual stromal tissue, it is possible to remove the cannula and then, by acquiring a longitudinal iOCT scan, observe the presence of a stromal pocket, which is visible as an hyperreflective line along the posterior stroma [30]. Alternatively, even if the cannula is not removed from the scleral pocket, a transversal iOCT scan across the width of the instrument can still allow for the visualization of a hyperreflective line extending laterally to the shadow of the cannula, similar to a “seagull wing” appearance (Figure 1). Once this line is identified, the measurement of the residual stromal bed can be performed with the caliper, which is usually integrated into the iOCT’s software, using the “seagull wing” as a reference.

The proper identification of the residual stromal thickness under the cannula is useful for predicting the probability of achieving the big bubble. In 2013, Scorcia and colleagues observed how the distance between the cannula tip and the DM was significantly smaller (90.4 ± 27.7 μm) in cases in which a big bubble was achieved than in a group of unsuccessful surgeries (136.7 ± 24.2 μm) [31]. Indeed, corneal surgeons routinely employing iOCT could corroborate their evaluation of stromal depth with actual real-time thickness measurements, deciding whether a repositioning of the cannula to a deeper stromal plane is advisable and weighing the higher chances of achieving a big bubble with the increased risk of perforation.

**Figure 2:**
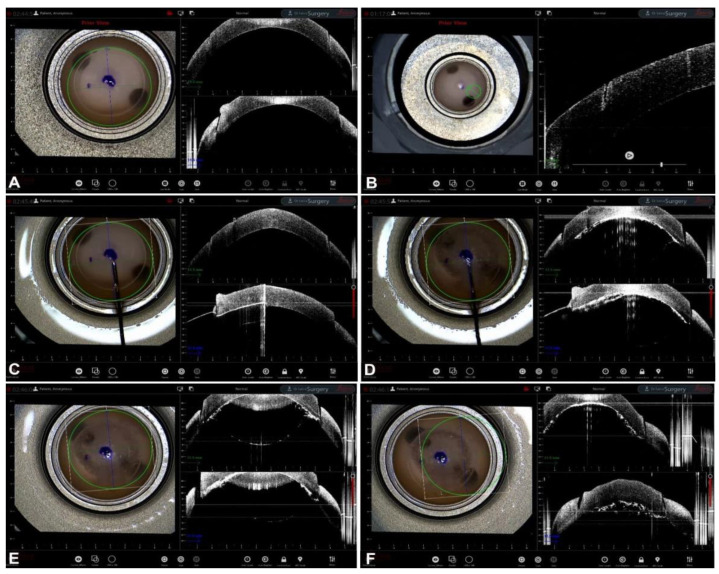
BB-DALK. (**A**) Stromal trephination. (**B**) Detail of the vertical hyperreflective band along the anterior stroma. (**C**) Insertion of the cannula. (**D**) Injection of air and bubble formation. (**E**) Bubble profile. (**F**) Detail of bubble and trephination.

The next step is the creation of the big bubble itself is to obtain a cleavage plan of the posterior stroma. The successful formation of the bubble is visualized on iOCT scans by observing the separation between the corneal stroma and DM, visible as a clear space between the internal concave surface and the external convex surface [32]. iOCT is capable of detecting sub-clinical big bubbles that are indistinguishable to the human eye in 40% of cases [33]. Moreover, iOCT could theoretically help to differentiate a type 1-BB from a type 2-BB by measuring the posterior wall of the bubble, which is made by pre-Descemetic stroma (the so-called Dua’s layer) and DM in type 1 and by DM only in type 2. The posterior wall of type 2 bubbles should therefore be thinner than the type 1 wall, even though this difference is in the order of a few microns and modern iOCT has not yet attained an image resolution capable of consistently differentiating the two bubbles [34]. In cases of formation of small air bubbles distributed over the whole stroma, iOCT helps in identifying each bubble, allowing the surgeon to puncture them with a sharp instrument (e.g., a 15-degree blade) to create a bigger cavity, thus rescuing the surgery and proceeding with the stromal dissection without the need of a conversion to PK [35] (Figure 2).

## 3. Guiding Manual Stromal Dissection DALK

Although BB-DALK is the preferred technique for performing anterior lamellar keratoplasty, there are few situations in which a bubble cannot be achieved or is associated with a high risk of perforation. Specifically, in cases in which corneal opacities do not allow for the visualization of the depth of the stromal involvement, or cases in which the bubble could easily provoke DM ruptures (e.g., radial keratotomies), the majority of corneal surgeons prefer to perform manual stromal dissection.

Intraoperative OCT could be helpful during the multiple surgical steps of DALK by stromal dissection. Firstly, iOCT permits the determination of the depth of the opacity and therefore the type of ALK to perform. Variations in corneal thickness at different points may be visualized in irregular or ectatic corneas, helping surgeons decide on a safe depth of initial trephination [7]. In other words, iOCT guides decision making with respect to the depth of trephination and prompts further eventual dissection.

iOCT is especially useful because it allows the surgeon to better control the incision depth and assess the uniformity of the dissection plane to optimize visual outcomes, especially when coaxial microscopy does not offer an excellent evaluation of the depth of a corneal incision [34,36]. In addition, iOCT could be very useful in assessing the residual stroma during dissection. Regarding the BB technique, the aim is to reach the pre-Descemetic plane, which guarantees a combination of good visual outcomes and a low risk of Descemet perforation. Hence, the optimal stromal bed should have a thickness of no more than 100 μm [24,33].

Moreover, the use of OCT enables surgeons to attempt manual dissection in the case of an emphysematous opaque cornea after a failed big bubble attempt: the opaque bubble layer hampers visualization during subsequent dissection; thus, iOCT can help surgeons perform a safe manual dissection and minimize the risk of DM perforation [37].

During manual stromal dissection in particular, sharp instruments inserted too deeply into the stroma can cause a DM rupture. In these cases, iOCT can detect the location of the rupture and guide further dissection. When a DM rupture is present, it can be advantageous to inject air into the anterior chamber to keep it formed. However, in cases of narrow iridocorneal angles, the air can be misdirected into the posterior chamber, causing an iris protrusion, which can be readily detected by the iOCT [38].

Once the proper plan is achieved, it is possible to proceed with the graft placement. In this step, iOCT might be useful in detecting residual interface fluid, allowing the surgeon to evacuate it by applying moderate massage on the corneal surface, leading to a reduced risk of a double anterior chamber in the postoperative period. After lamellar dissection, iOCT aids in the assessment of graft thickness, graft–host apposition, and interface regularity, as well as in the evaluation of any residual stromal pathology or disparities in the graft–host sizing [35].

Finally, iOCT can aid in the management of post-DALK Descemet’s membrane detachment (DMD) by determining the localization of maximal DMD, managing an iOCT-assisted injection of isoexpansile gas SF6 (BVI Medical, Waltham, USA) into the anterior chamber, guiding the location for venting incisions to drain the interface fluid, and confirming the apposition of the grafts [39].

## 4. Guiding Ultra-Thin Descemet Stripping Automated Endothelial Keratoplasy

Descemet Stripping automated endothelial keratoplasty (DSAEK) is one of the leading procedures for the management of corneal endothelial dysfunction [40]. Compared to PK, DSAEK has faster recovery times and better final visual and contrast acuity with less surgically induced astigmatism and fewer higher-order aberrations [41,42]. Graft rejection is also much more frequent in PK compared to DSAEK [43]. Some intra- and post-operative challenges specific to endothelial keratoplasty exist, such as graft visualization, graft apposition and dislocation, and interface complications [9]. However, iOCT can aid the corneal surgeon during every step of the DSAEK procedure, addressing each of these challenges [44].

**Figure 3:**
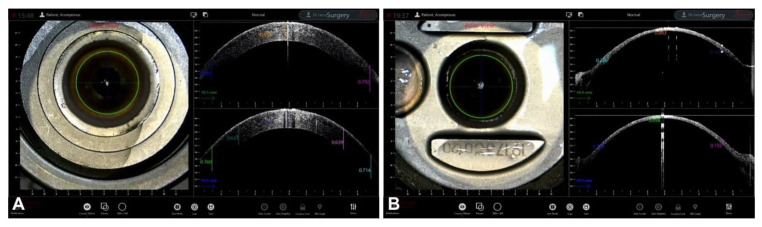
DSAEK preparation (**A**) Pre-cut thickness. (**B**) Post-cut thickness.

During graft preparation, either one or two consecutive passes of a microkeratome blade allow for the creation of a very thin lenticule in a procedure known as UltraThin-DSAEK (UT-DSAEK), which has become the standard modern approach to DSAEK [40,45,46]. In cases in which this preparation is carried out in the operating room rather than at the eye bank, iOCT allows the surgeon to easily check the residual stromal thickness, the smoothness of the cut, and the regularity of the UT-DSAEK lenticule [47,48,49,50,51] (Figure 3).

The iOCT can also be useful for visualizing the DM during descemetorhexis, as well as for locating DM residues and guiding their removal under direct visualization, even in cases of poor visibility due to corneal edema or scarring [9,52,53,54]. With the help of iOCT, DSAEK can be successful in patients affected by a clinically opaque cornea, which would otherwise be treated with a penetrating keratoplasty [52].

One of the most significant contributions of iOCT comes at perhaps the most crucial step of DSAEK: the apposition of the DSAEK lenticule to the recipient stroma. The assessment of the graft attachment and the evaluation of the residual interface fluid between the graft and the recipient stroma are readily available to the surgeon using iOCT. The routinary use of iOCT could, for instance, supersede the use of venting incisions, which have been developed to preemptively drain any potential residual fluid in the graft–stroma interface. Under direct iOCT visualization, residual fluid can be expressed out of the interface through focal manipulation and sweeping of the cornea [44,53,55]. Residual fluid is associated with early graft nonadherence and can result in textural interface opacities due to delayed gap closure, precipitated solutes, the retention of a viscoelastic substance, or lamellar irregularities caused by delayed adhesion or uneven matching of lamellar fibrils [56,57].

The iOCT can also aid in positioning the graft in cases of uneven posterior corneal surfaces, such as in cases of a previous penetrating keratoplasty in which the posterior lip of the trephination margin could hamper the successful attachment of a DSAEK graft. If a focal irregularity of the posterior corneal surface is detected, the graft can be recentered far from the irregular area [44].

**Figure 4:**
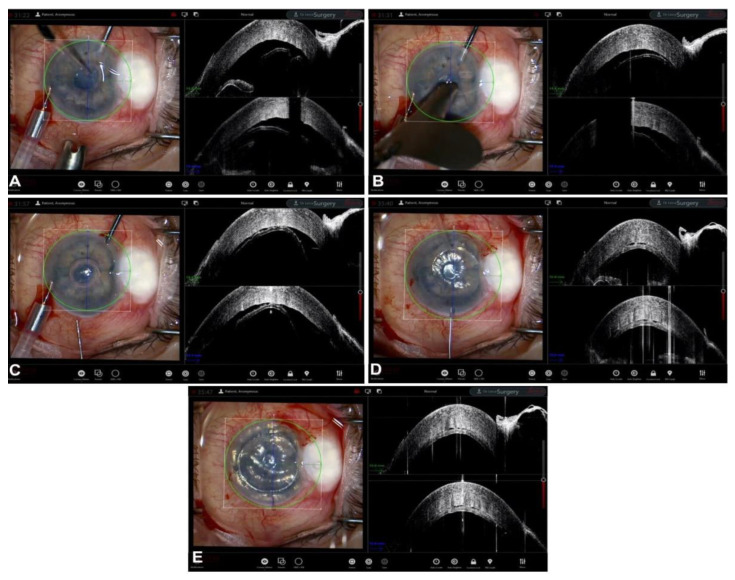
DSAEK. (**A**) Pull-through insertion. (**B**) Deployment. (**C**) Partial AC filling. (**D**) Interface fluid. (**E**) Apposition and AC filling.

Finally, iOCT’s role could be even more prominent in times in which nanothin (<50 μm) DSAEK is gaining popularity: in complex eyes at a higher risk for rejection and graft detachment, nanothin DSAEK offers comparable results to Descemet membrane endothelial keratoplasty (DMEK) with lower rates of complication, and its results are enhanced by the use of iOCT for correct positioning and orientation [58,59] (Figure 4).

## 5. Guiding Descemet Membrane Endothelial Keratoplasty

Although it is steadily gaining popularity, Descemet membrane endothelial keratoplasty (DMEK) is still considered to be a difficult technique characterized by a steep learning curve [60,61,62]. iOCT can facilitate the procedure, helping the expert and the novice surgeon alike [44,63,64,65,66].

One of the most useful applications of iOCT in DMEK surgery is the possibility of confirming the correct orientation of the graft in real time, with the endothelial side facing the anterior chamber [37,63,65,66,67,68]. The graft can be delivered with the endothelium folded either outwards, which represents its natural scrolled configuration, or inwards. In the inwards configuration, the membrane is folded in the opposite way to its natural scrolling tendency but it requires minimal time for unfolding inside the eye since it is perceived as an unnatural conformation [69,70,71]. In either cases, the unfolding of the graft can be monitored with iOCT even in cases of cloudy corneas, and the correct orientation can be confirmed [64]. As strategies to mark the tissue can result in endothelial damage or are inherently subtractive of endothelial cells, the possibility of checking the orientation of the graft indirectly could improve the long-term survival of the transplant [61,72,73].

At present, the orientation must be confirmed qualitatively by the surgeon based on the rolling and unfolding properties of the graft, as shown by iOCT [65]. However, the semi-automated or automated classification of graft orientation will most likely be integrated with iOCT: in 2013, Steven and colleagues correlated the intraoperative scrolling properties of DMEK grafts with donor age by mathematically describing the average curvature of the grafts, based on an image analysis of iOCT frames [63]. Building on this concept of curvature in relation to orientation of the graft, the deep learning segmentation of DMEK grafts has been applied to individual iOCT frames to automatically recognize the free-floating graft, compute its local curvature, and predict its orientation, with results comparable to a cornea specialist [68,74].

**Figure 5:**
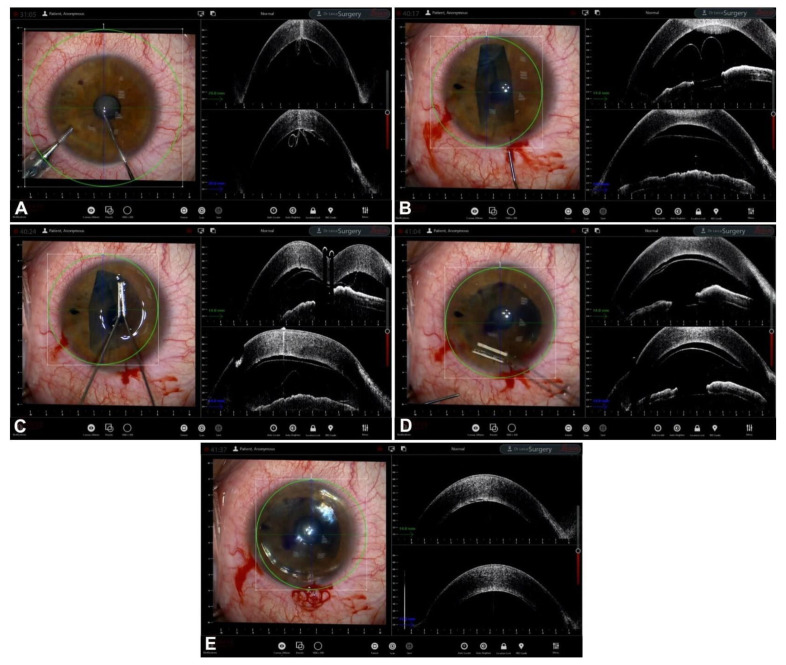
DMEK. (**A**) Deschemetorexhis. (**B**) Graft scroll. (**C**) Dirisamer maneuver. (**D**) Graft orientation and interface fluid. (**E**) AC filling.

As in DSAEK surgery, iOCT during DMEK can be helpful not only to check the configuration and orientation of the graft but also to visualize areas of synechiae, remnants of Descemet membrane, the presence of fluid or folds at the graft–stromal interface, and to center the graft [63,64,75,76]. Finally, iOCT can also help in the preparation of the graft as well as in teaching novice surgeons. In fact, iOCT has been used to quantitatively assess the quality of donor corneas and pre-stripped grafts and to monitor each step of the stripping procedure [47,48]. Likewise, wet labs equipped with microscope-integrated iOCT allow expert surgeons to monitor the progression of their pupils and correct them accordingly [77]. (Figure 5).

## 6. Guiding Mushroom Penetrating Keratoplasty

The idea of asymmetrically shaping grafts in order to take advantage of the specific features of each layer of the cornea is not new [26,78,79]. The term “mushroom PK” was created in the 1950s, and its purpose is to minimize the replacement of the recipient’s healthy endothelium while maintaining a large diameter of the superficial, refractive part of the graft [78,80]. This combines the benefits of lower incidences of induced astigmatism and rapid postoperative healing while minimizing endothelial cell loss and the risk of immunologic rejection and graft failure [80,81,82,83].

A variety of technique for preparing mushroom grafts have been described, from manual trephination to femtosecond-laser-assisted mushroom PK [82,84]. In 2005, Busin and colleagues created a two-piece mushroom PK by splitting the donor cornea in two with the help of a microkeratome and then punching the anterior and posterior lamellae to different sizes [80,81,85].

**Figure 6:**
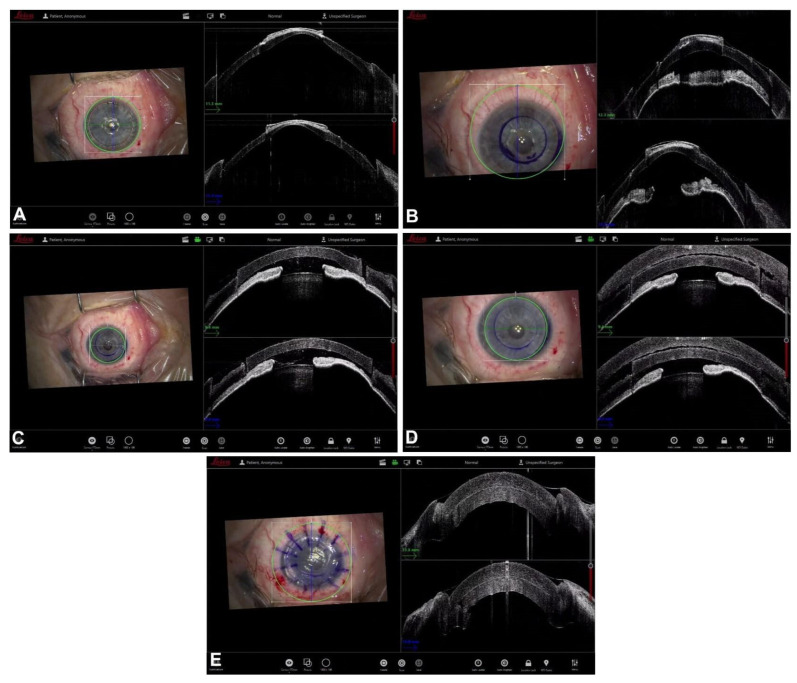
Mushroom PK. (**A**) Dissection. (**B**) Trephination. (**C**) Internal lamella placement. (**D**) External lamella placement. (**E**) Interface.

Although no publication in the literature thus far has focused on the use of iOCT in mushroom PK, its unique ability to examine the profile of asymmetric cuts and their relationship with the recipient tissue in real time can surely be of great help to the surgeon. In Figure 6, various steps of an iOCT-guided mushroom PK surgery are displayed. It is immediately obvious how iOCT can contribute and improve this complex surgery by visualizing the relationship between the graft layers and host tissue, allowing the surgeon to check for the correct positioning of the transplant. 

Mushroom PK is only one of the possible asymmetric graft configurations; the most well-known other configurations are the top-hat, the anvil, and the zig-zag configurations. Each exploits the benefits of their particular shape and is tailored to the need of the individual diseased recipient corneas [86,87]. Asymmetric configurations, and in particular mushroom PK, are particularly suited to cases of thin, one-sided recipient corneas such as in very peripheral ectasias [82]. In fact, mushroom PK allows the surgeon to address the mismatch in thickness between the graft and the host by leaving behind a non-visually significant step between the two posterior surfaces while guaranteeing a continuous and smooth interface at the anterior refractive surface [75,82]. iOCT can clearly aid the surgeon in the positioning of mismatching surfaces in mushroom PK. Another possibility in mushroom PK that can greatly benefit from real-time positioning thanks to iOCT is the differential centration of the anterior and posterior lamellae, with the former usually being centered on the corneoscleral limbus and the latter on the visual axis, as described by Busin in 2005 [85].

## 7. Future Developments

To date, the most important limiting factor of a wider adoption of iOCTs is the entry cost of the device. The use of OCT in the operating room has transitioned from handheld OCTs to microscope-mounted OCTs and finally to microscope-integrated iOCTs, which have largely superseded earlier devices. This means that the entry cost for a state-of-the-art iOCT also includes, in more cases than not, the purchase of an integrated microscope.

The DISCOVER and PIONEER studies provided solid evidence of the utility of iOCT in modern eye surgery, but no large clinical randomized trial has definitely proven the superiority of iOCT to microscopy alone [9,11].

It is reasonable to think that with the increased adoption of these devices, their cost will also be mitigated [44]. The wider diffusion of this technology would allow its application in even more types of eye surgery, such as refractive surgery, and in the SMILE technique first of all. Indeed, the iOCT’s aid permits the better visualization of the stromal lenticule and confirms the absence of cap–lenticule adhesion [88].

Furthermore, iOCTs are still evolving devices and, in addition to decreasing costs, their technical specifications are also expected to improve. Available iOCT devices currently employ a central wavelength of 840–860 nm and are capable of axial resolutions between 2.5 and 5 µm in the order of high-resolution posterior segment OCT [89]. However, their lateral resolution and A-Scan rate, arguably more relevant to corneal lamellar surgery, are currently working at 15–30 µm and 10–36 kHz, respectively. As a result of the latter, the acquisition of volume scans takes several seconds to complete and is not feasible during active surgical steps. Instead, continuous, high-resolution, single-line B-scans are usually employed to track the action [90]. On the other hand, increasing the lateral resolution could improve the visualization of finer details of the corneal anatomy and pathology [90].

Further developments of iOCT would also include even larger fields of view than what is currently available (up to 16–30 mm, depending on the device), which could allow the surgeon to easily monitor the entire surgical field, from the center of the cornea to the periphery of the anterior chamber, in one glimpse. Moreover, improvement of the automated tracking of surgical instruments and iOCT autofocusing on the corneal structure of interest, which may escape the field of view during surgical movements, are warranted [91].

Finally, with the goal of creating an all-in-one platform, microscope-integrated iOCT could be augmented with automated image analysis, providing useful insights on what is happening in the surgical field in real time, such as the analysis of graft orientation [68], or with the concurrently developing 3D imaging and robotically assisted surgery [92].

## Data Availability

No new data were created or analyzed in this study. Data sharing is not applicable to this article.
